# Variability in the Nutritional Adequacy of Primary School Meals in Zagreb: The Role of School Environmental Factors

**DOI:** 10.3390/nu18213709

**Published:** 2026-11-09

**Authors:** Ana Ilić, Ivana Rumbak, Ružica Brečić, Martina Bituh

**Affiliations:** 1 Department of Nutrition, Food Quality and Safety, Faculty of Food Technology and Biotechnology, University of Zagreb, Pierottijeva 6, 10000 Zagreb, Croatia; ana.ilic@pbf.unizg.hr (A.I.); martina.bituh@pbf.unizg.hr (M.B.); 2 Department of Marketing, Faculty of Economics and Business, University of Zagreb, Trg J. F. Kennedy 6, 10000 Zagreb, Croatia; rbrecic@efzg.hr

**Keywords:** school food system, school meals, Croatia, primary school, meal quality

## Abstract

Background/Objectives: Although investments are being made in school meals worldwide, their quality remains problematic due to financial, knowledge-related, or logistical factors. The aim of this study was to examine the nutritional adequacy of school meals in Zagreb (Croatia) and to determine whether the energy adequacy differs depending on selected environmental factors such as school location and neighborhood poverty rate, type of meal service system, staffing, menu planning, and participation in school-based nutrition initiatives and programs. Methods: The study was conducted in 14 primary schools in the city of Zagreb during the 2016/2017 and 2017/2018 school years. A total of 2469 daily menus were collected (2379 breakfasts, 2376 lunches, and 2103 snacks). Menus and information on environmental factors were obtained through on-site audits, direct dialogs with staff, and school curricula. The energy and macronutrient content of school meals was analyzed using Prehrana software version 1.0 and evaluated according to national guidelines. Results: The energy and macronutrient content varied greatly among schools. In 75% of schools, the average energy and macronutrient content of breakfasts exceeded 110% of recommendations, while 70% of schools averaged less than 90% of recommendations for energy and macronutrients in lunches. Considering energy and macronutrient content, three breakfasts, 38 lunches, and one snack met all four recommendations. Among the school-level environmental factors observed, the proportion of energy-adequate meals differed significantly by kitchen type, number of on-site-prepared meals, and neighborhood poverty for lunches, as well as school garden presence for breakfasts. Conclusions: The findings reveal differences in the nutritional adequacy of school meals across primary schools in Zagreb and may help guide future improvements in school food system guidelines and practices.

## 1. Introduction

School food systems play a crucial role in the lives of school-age children, as they can provide up to three meals during school hours [1,2]. These meals must be nutritionally balanced to support healthy growth and development [2,3] and promote long-term healthy eating habits [4,5,6]. According to the available literature, children who participate in school meal programs (breakfast and/or lunch) have better overall diet quality and variety and more adequate nutrient intake. Moreover, they are more likely to consume nutrient-rich foods such as fruits, vegetables, whole grains, and dairy products [7,8,9,10,11,12,13,14]. Furthermore, consuming school meals can reduce disparities in overall diet quality among children from different income backgrounds [14,15]. Beyond the short-term benefits of meeting energy and macronutrient needs, school meal programs may shape children’s food choices, taste preferences, and eating behaviors [16]. In addition to these behavioral benefits, school meal programs may have positive long-term implications for health, cognitive outcomes, and academic performance [17,18]. Promoting and adopting healthy dietary habits in children may be linked to health outcomes in adulthood [19]. For example, a recent study of Italian university students found that greater adherence to the Mediterranean diet was associated with fewer depressive symptoms [20]. Nevertheless, the benefits of school meal programs depend on their nutritional adequacy and quality. School food systems continue to struggle with challenges related to meal adequacy, most commonly low energy and macronutrient content, as well as high levels of sodium, added sugar, and saturated fatty acids [11,13,15,21,22,23]. To fully reap the benefits of school meals, each country needs to develop its own nutrition policies and guidelines for organized school food systems and ensure they are effectively implemented in schools [2,24]. Possible challenges to implementing these policies and guidelines in the school food system include limited stakeholder awareness of the need to procure highly nutritious food, insufficient funding, shortages of kitchen staff, and inadequate equipment [25,26,27].

In Croatia, primary schools are required to provide school meals for students in accordance with the Act on Education in Primary and Secondary Schools and the State Pedagogical Standard of the Primary School Education System [28,29]. As children may be in school for up to 8 h, schools serve one to three meals: breakfast, lunch, and a snack (served in the afternoon). The school schedule and lesson length determine which meal types are available to children in primary schools, and parents decide whether to enroll their children in some or all of these options. In the 2021/2022 school year, 72.2% of schools offered only breakfasts, 32.5% offered breakfasts and lunches, and 29.1% offered only lunches. These proportions were similar in the 2016/2017 to 2019/2020 school years before the COVID-19 pandemic [30]. Before the 2022/2023 school year, meals were co-financed by state, regional, and local self-government units, with full subsidies for children from socially disadvantaged families, families without income, and children of war veterans [31]. From the 2022/2023 school year onward, the national government introduced universal school meal funding, providing €1.33 per child who regularly attends school [32].

According to legislative frameworks [28,29], local governing authorities (school founders) and school administration have the responsibility of organizing the school food system. This includes providing meals in accordance with the standards launched by the Ministry of Health of the Republic of Croatia in 2012 [33]. In 2013, to ensure high-quality implementation of these standards and improve school food systems, the Ministry of Health of the Republic of Croatia published the National Guidelines for the Nutrition of Students in Primary Schools [34]. Furthermore, to improve the quality of school meals in Croatia, three voluntary school-based nutrition initiatives and programs are being implemented: Živjeti zdravo, School Scheme, and Kaufland Fruit and Vegetables [35,36,37]. School Scheme and Kaufland Fruit and Vegetables provide supplemental procurement of fruit, vegetables, milk, and dairy products [36,37], while Živjeti zdravo (2016–2022) focuses on auditing school menus according to the standards and offers menu changes with accompanying meal recipes [35]. However, data on the nutritional adequacy of school meals in primary schools in Croatia remains limited.

In this study, we address two key research questions to expand the current literature and identify potential areas for improvement or reorganization to enhance the adequacy of school meals within current guidelines. Therefore, we evaluate the nutritional (energy and macronutrient) adequacy of different types of school meals offered in 14 primary schools in the city of Zagreb (Croatia). Furthermore, we conduct exploratory analyses to examine the association between meal energy adequacy and selected school-level environmental factors (e.g., school location, school garden, kitchen type, meal serving systems, kitchen staffing, and neighborhood poverty status).

## 2. Materials and Methods

### 2.1. Study Design and School Selection

This cross-sectional study used data collected within the framework of the Horizon 2020 project “Strengthening European Food Chain Sustainability by Quality and Procurement” (Strength2Food, H2020-SFS-2015-2, contract number 678024), in accordance with the project’s standardized methodology. To achieve the objectives of the Strength2Food project, the first step of the sampling strategy included schools with educational gardens located in different city areas. Of the eligible public schools, 7 agreed to participate in the project. In the second step, a matched quasi-randomized sampling protocol was implemented using a custom statistical randomization algorithm (programming language C# and Oracle Express database with PL/SQL), with the following selection criteria: non-garden primary schools; location in different city areas but similar to those of schools with gardens; and willingness to participate, as several schools were in acceptable city areas. For the Strength2Food project, to assess group differences between schools with and without gardens, the sample was defined as 14 schools (ANOVA ω2 ≥ 0.14). Although the original sampling protocol was designed around garden-matching, these 14 schools represent 13% of public schools in the city of Zagreb (*n* = 107) at the time of data collection. The collected dataset provides a comprehensive view of annual school menus and organizational and environmental factors, offering an exploratory link between nutritional adequacy and these factors. By including primary schools from different areas of the city, this sample provides a robust and realistic picture of the school food system in the city of Zagreb.

In each school, the principal gave consent for participation in the project and for access to information about the school and its dissemination. Approvals for implementation of the Horizon 2020 project in elementary schools were obtained from the Ethics Committee of the Institute for Medical Research and Occupational Health (no. 100-21/16-8), the Croatian Ministry of Science and Education, and the Education and Teacher Training Agency (no. 602-01/16-01/00388).

### 2.2. Assessment of School Meal Nutritional Adequacy

**Figure 1:**
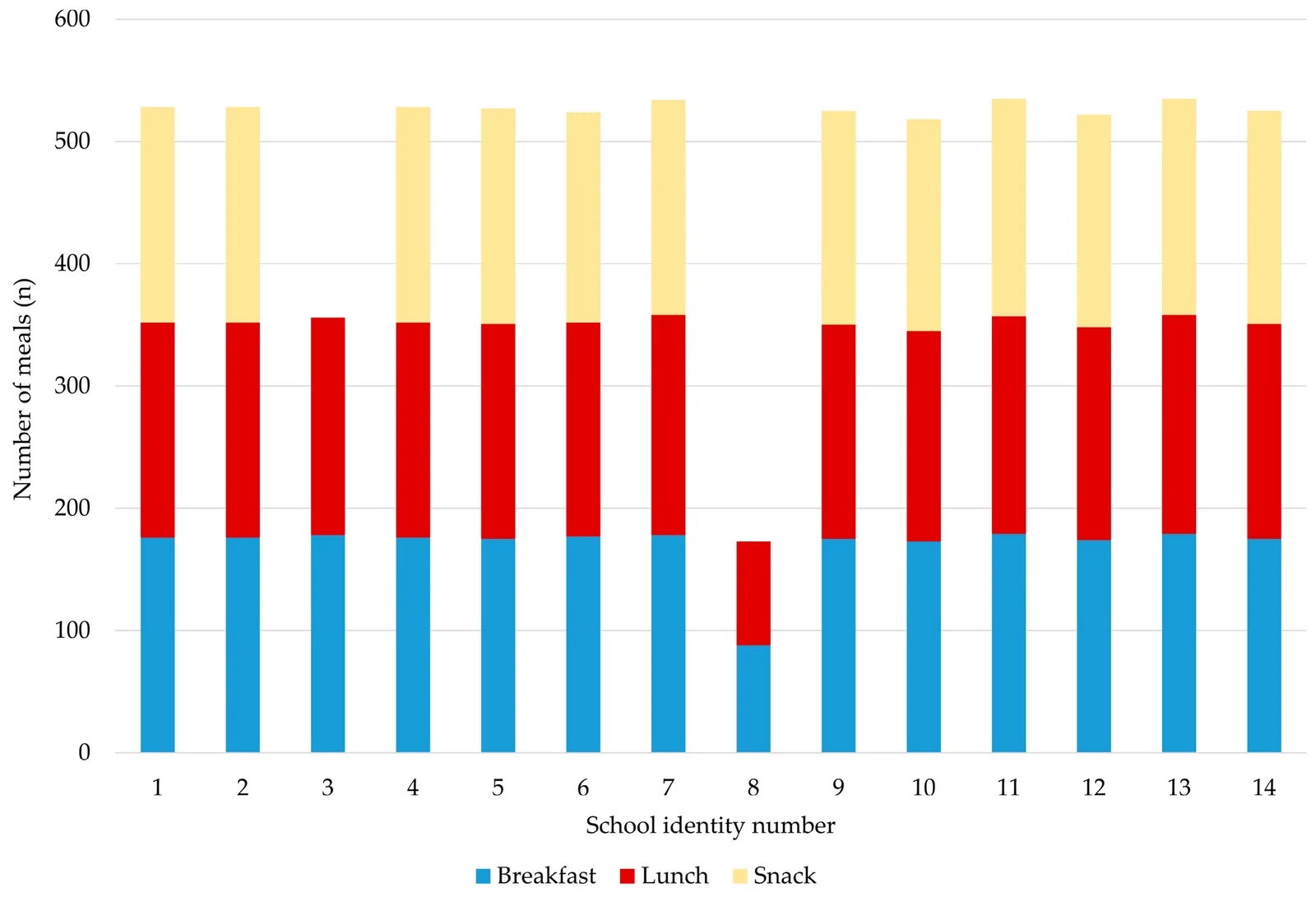
Type and total number of meals offered in annual school menus across primary schools (*N* = 14) in Zagreb.

Data collection included annual menus from 14 primary schools covering one school year. Menus from ten schools were collected in the 2016/2017 school year and from the remaining four schools in the 2017/2018 school year due to data collection logistics. According to school staff interviews, no organizational changes occurred in these four schools between the two school years. Therefore, each school’s data represent one annual menu cycle without repetition across school years. In total, 2469 daily menus were collected, comprising 2379 breakfasts, 2376 lunches, and 2103 snacks. Detailed information on the number of offered meal types across 14 schools is shown in Figure 1.

The schools’ annual menus were collected by school staff (principal, secretary, accountant, or kitchen manager), while the recipes (food weight for preparation, preparation method, and product brands) for all meals were collected in an interview with the school chef. The research team calculated all recipes as one serving portion for the children. To estimate energy and macronutrient values, all meals were analyzed using Prehrana software (v. 1.0, Infosistem Plc., Zagreb, Croatia). The Prehrana software contains the chemical composition of foods and beverages from the Croatian national composition tables [38] and is supplemented with the Danish food database [39]. Additionally, the program is supplemented with data on energy and nutrient values from food labeling for each food product on the market that is not included in the Croatian national composition tables.

The adequacy of energy and macronutrient content of each meal was assessed according to the National Guidelines for the Nutrition of Students in Primary Schools [33,34]. In the national guidelines, recommendations are expressed as daily intakes of energy and macronutrients according to age (7–9 years, 10–13 years, and 14–18 years) and sex. To standardize the recommendations for the analysis, the daily recommendations for children aged 7–9 years were calculated as the average for both sexes. This age group was chosen because most children in lower primary school grades (grades 1–3) participate in the school meal program and consume several school meals per day. In addition, national guidelines define recommendations for energy at both the daily level and per individual meal, whereas recommendations for macronutrients are define only at the daily level. Therefore, meal-specific macronutrient recommendations were calculated proportionally based on the daily energy contribution of each meal type, as presented in Table 1. Meal energy and macronutrient adequacy were evaluated against calculated recommendations using classification criteria based on national guidelines: energy, protein, and fat had two-sided thresholds (inadequate: below or above the threshold), while carbohydrate had a one-sided threshold (inadequate: below the threshold).

### 2.3. Environmental Factors

One aim of the present study was to conduct exploratory analyses to examine the association between meal energy adequacy and environmental factors. In this analysis, energy was selected as the metric of nutritional adequacy because it is expressed at the meal level in the guidelines and was not calculated for this study. The observed environmental factors and their descriptions are shown in Table 2. Information on environmental factors was collected through an on-site observational audit. For each school, the research team observed the school food system organization and collected details during direct dialog with school staff (principal, secretary, accountant, kitchen manager, and school cook). This observation provided an objective metric regarding the organization of the school food system without collecting personal data from school staff. Finally, the observation was completed by reviewing the school curricula to determine whether the schools participated in additional nutrition programs, health promotion projects, or initiatives.

### 2.4. Statistical Analysis

Descriptive statistics (minimum, 25th percentile, median, mean, 75th percentile, and maximum) were used to show the energy and macronutrient values of school meals in each school. The extent to which the school meals in each school met the recommendations for energy and macronutrient content was presented as the mean, as this variable was normally distributed according to the Shapiro–Wilk test. Categorical variables were presented as frequencies or percentages. Differences in the energy and macronutrient values of school meals among 14 schools were evaluated using the Kruskal–Wallis test. Post hoc pairwise comparisons were conducted using Dunn’s test with a Bonferroni correction to adjust for multiple testing. Adjusted *p* values for all pairwise comparisons are provided in the Supplementary Materials (Tables S1–S3). To account for the hierarchical structure of the data, environmental factors were evaluated at the school level (*N* = 14). For each school, the proportion of energy-adequate meals were calculated across each meal type. Differences in meal energy adequacy across environmental factors were analyzed using the Mann–Whitney U test and the Kruskal–Wallis test. Statistical significance was set at *p* < 0.05 for all statistical procedures. All statistical analyses were performed with IBM SPSS Statistics, v. 23.0, released in 2015 (IBM SPSS Statistics for Windows, Armonk, NY, USA: IBM Corp.). Graphics were created using IBM SPSS Statistics, v. 23.0, and Microsoft Office Excel 2016 (Microsoft, Seattle, WA, USA).

## 3. Results

**Figure 2:**
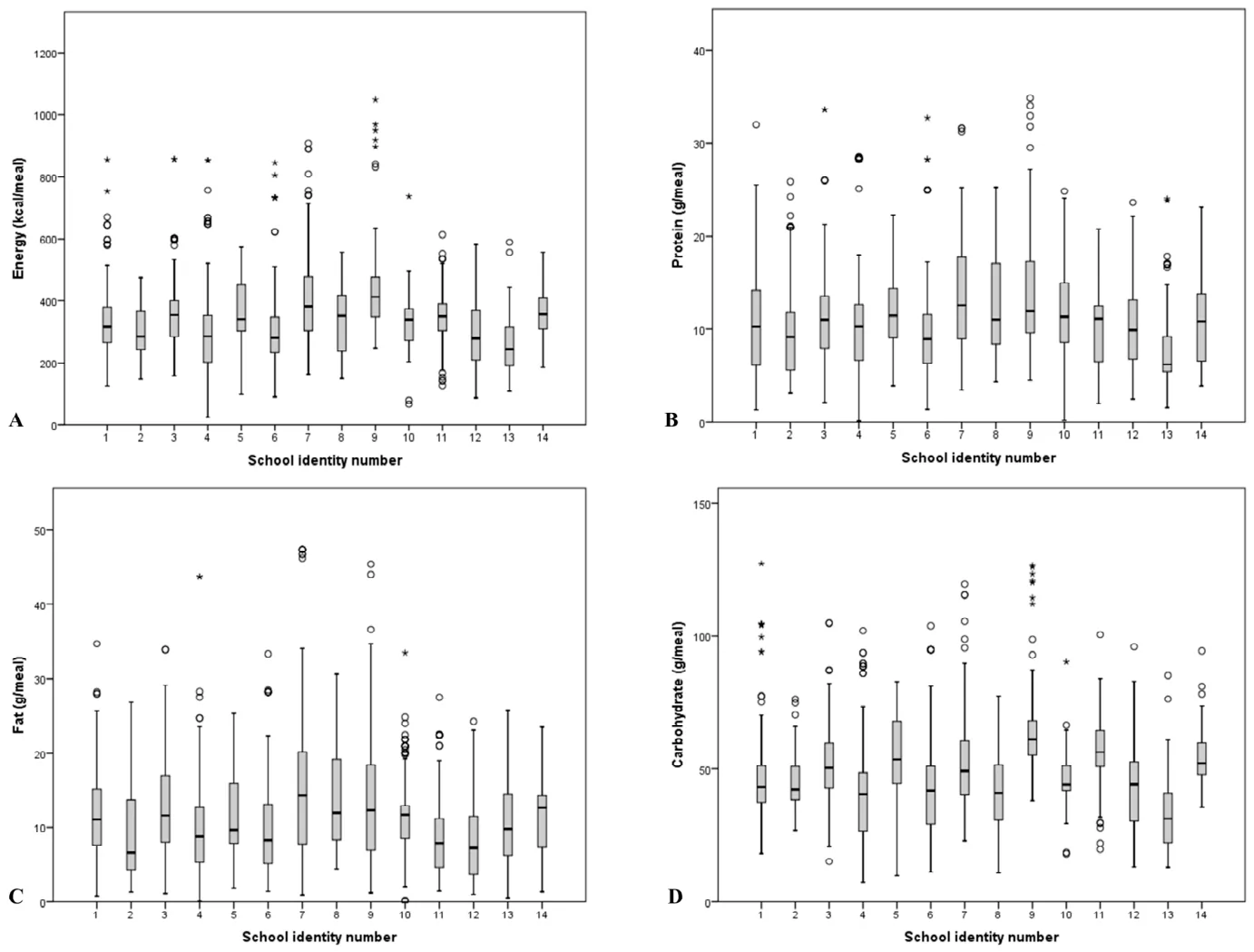
Distribution of energy (**A**), protein (**B**), fat (**C**), and carbohydrate (**D**) content in offered breakfasts from annual school menus in selected primary schools (*N* = 14) in Zagreb.

**Figure 3:**
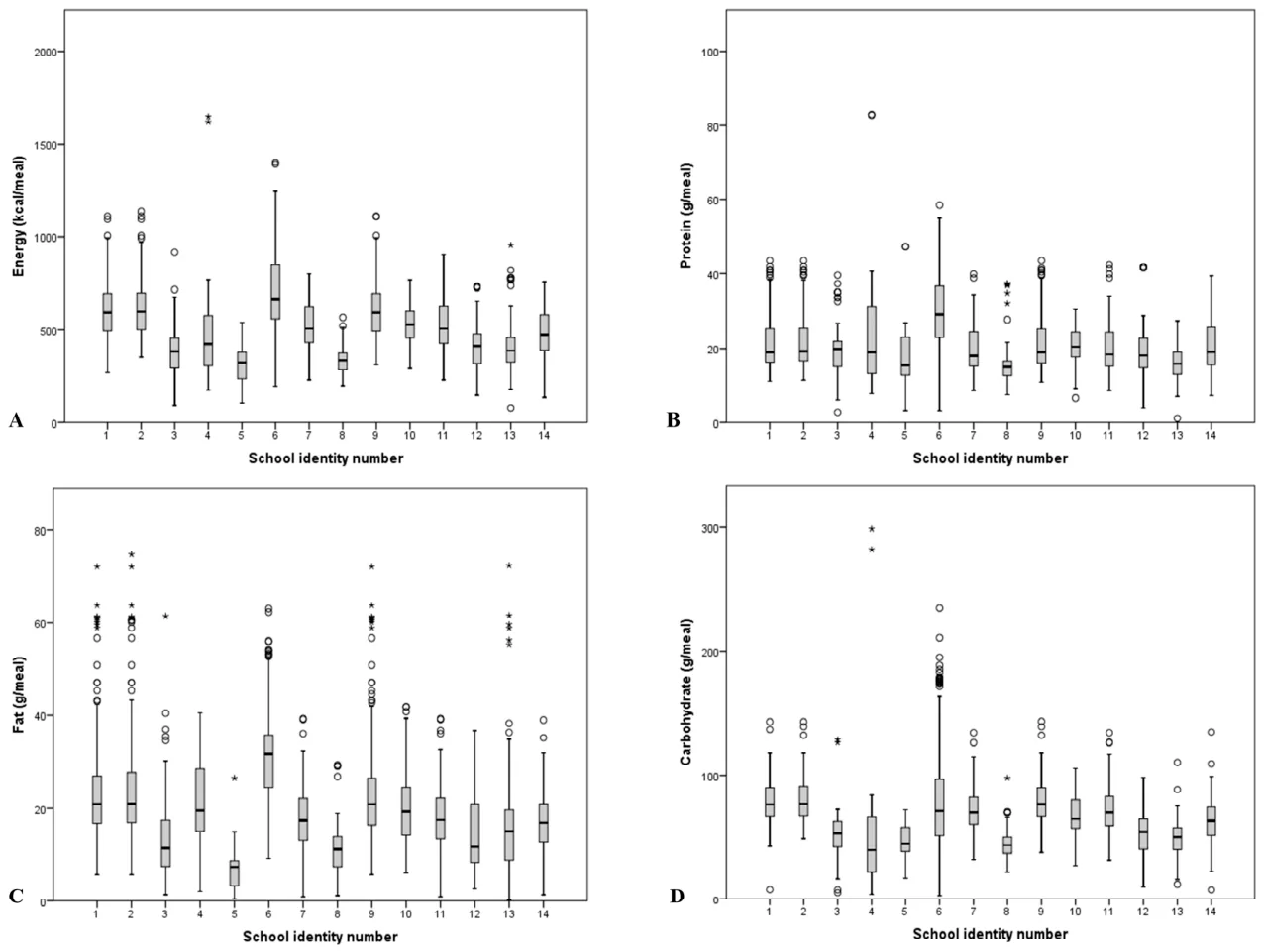
Distribution of energy (**A**), protein (**B**), fat (**C**), and carbohydrate (**D**) content in offered lunches from annual school menus in selected primary schools (*N* = 14) in Zagreb.

**Figure 4:**
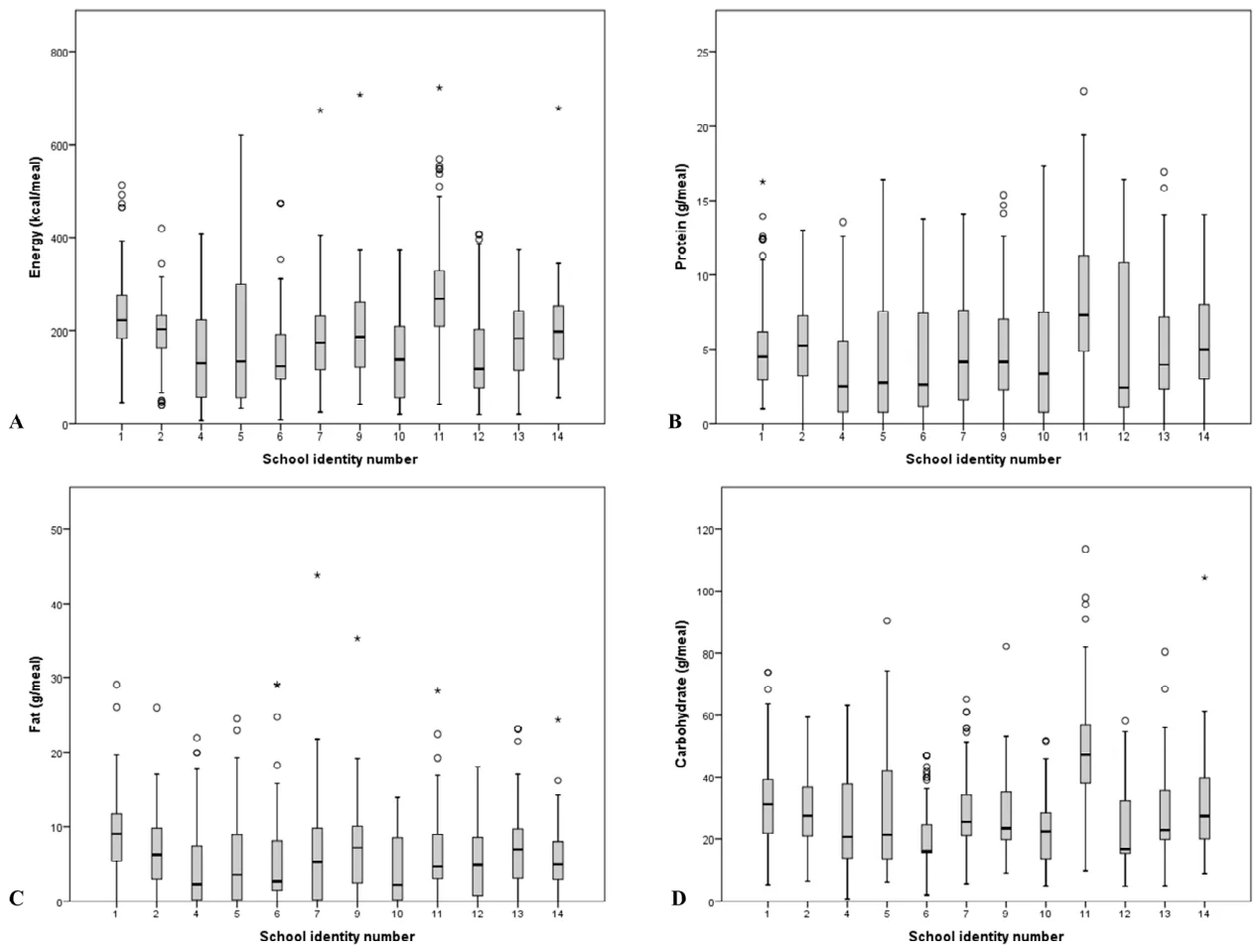
Distribution of energy (**A**), protein (**B**), fat (**C**), and carbohydrate (**D**) content in offered snacks from annual school menus in selected primary schools (*N* = 14) in Zagreb.

Variations in the energy, protein, fat, and carbohydrate values of breakfasts, lunches, and snacks are shown in Figure 2, Figure 3 and Figure 4. According to the Kruskal–Wallis test, there were significant differences in the energy and macronutrient content of all three meals among the schools (*p* < 0.001). For detailed information, post hoc analyses were conducted using Dunn’s test, and the differences are presented in Supplementary Tables S1–S3.

**Figure 5:**
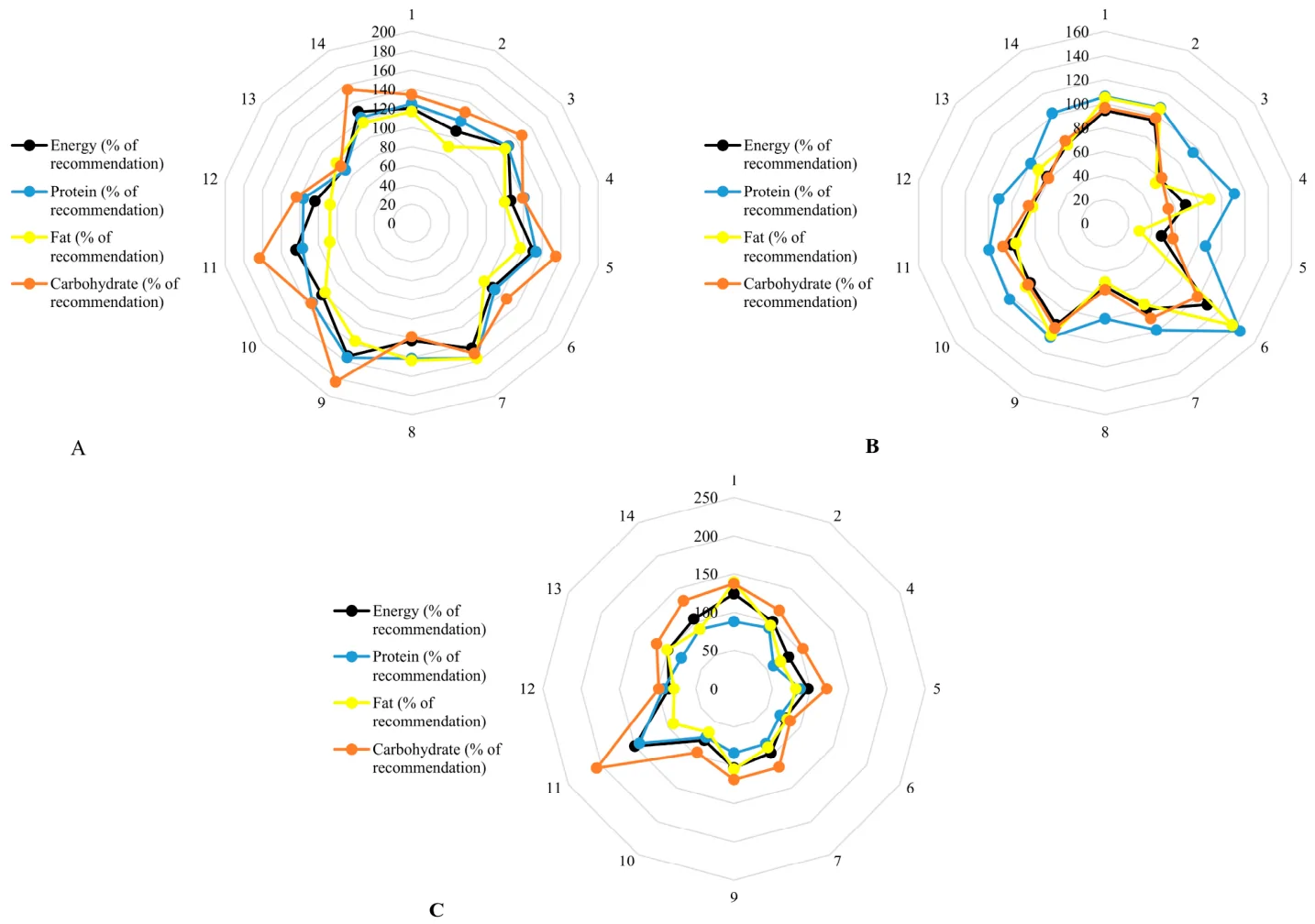
Compliance of offered breakfasts (**A**), lunches (**B**), and snacks (**C**) from annual school menus in selected primary schools (*N* = 14) in Zagreb with National Guidelines for the Nutrition of Students in Primary Schools in energy and macronutrient content.

The proportions of school meals that met the recommendations from the National Guidelines for the Nutrition of Students in Primary Schools for required energy and macronutrient content across all three school meals are shown in Figure 5. Most schools exceeded the recommended energy, protein, and carbohydrate content for breakfasts, with 50% of the schools also exceeding the recommendation for fat content. Conversely, almost all schools were below the thresholds for energy, fat, and carbohydrates in lunches, and 50% of schools were above the threshold for protein. For snacks, compliance with recommendations for energy, protein, and fat varied across schools, whereas carbohydrates were within the recommendations. One school (ID 11) stood out by providing snacks with average values that exceeded recommendations for energy by 50% and for protein by 43%.

**Figure 6:**
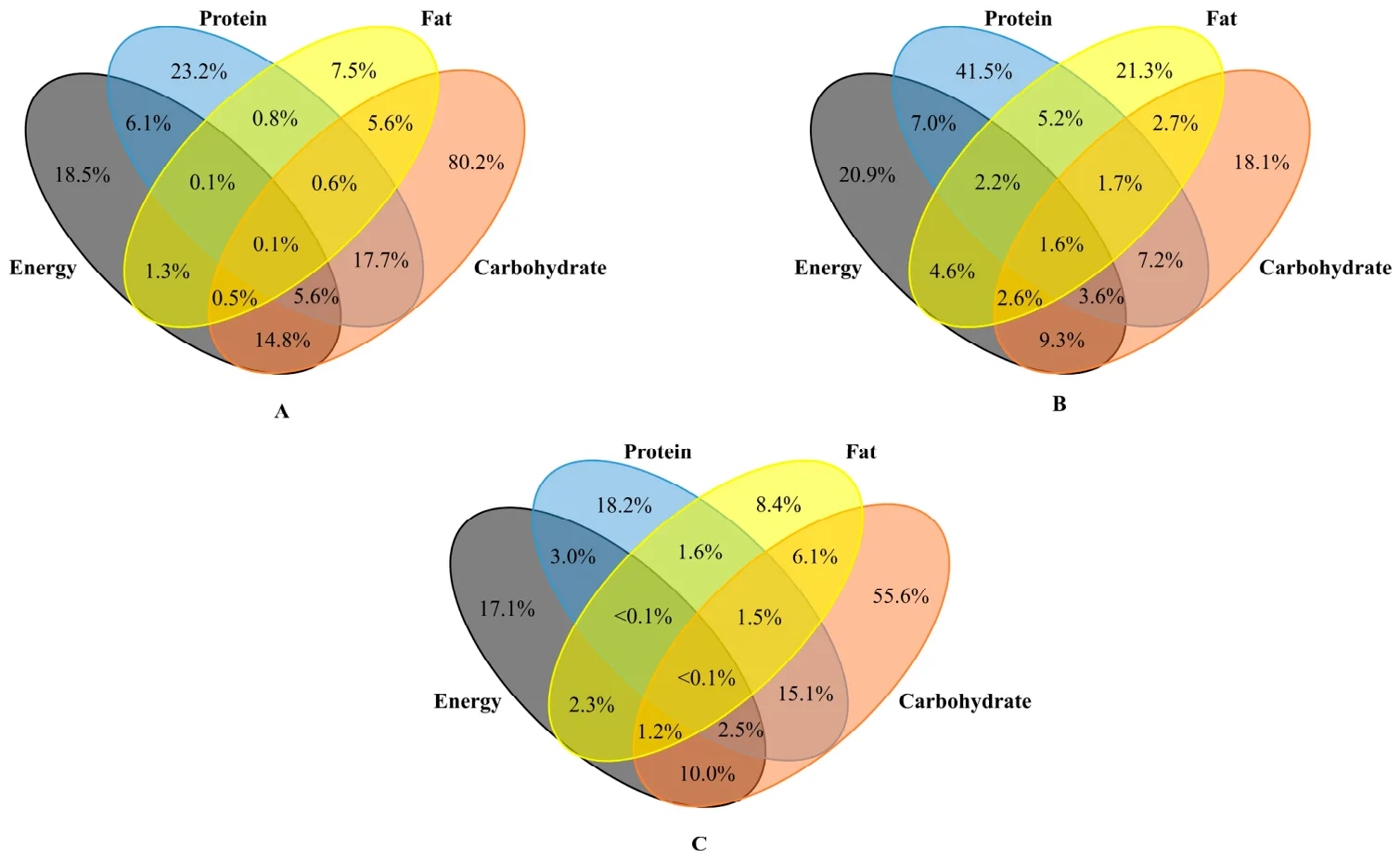
Venn diagrams illustrating the multi-criteria compliance of offered breakfasts (**A**), lunches (**B**), and snacks (**C**) from annual school menus in selected primary schools (*n* = 14) in Zagreb with recommendations for energy (gray), protein (blue), fat (yellow), and carbohydrates (orange). Values are presented as the percentage of evaluated meals.

Furthermore, Figure 6 synthesizes overall multi-criteria compliance across all three school meals. Notably, only 0.1% (*n* = 3) of breakfasts had adequate energy and macronutrient content, and the lowest proportion of school breakfasts had adequate fat content (7.5% of breakfasts). Of all school lunches, only 1.6% (*n* = 38) met all four recommendations, and the carbohydrate content was the least often met (18.1% of lunches). Only one school snack out of all 2103 had adequate energy and macronutrient content. Of the school snacks, at least 8.4% achieved an adequate amount of fat.

Finally, we examined environmental factors that were hypothesized to be associated with nutritional adequacy of school meals, taking into account the energy adequacy of the meals. Of the 12 potential factors, 10 were included in the exploratory bivariate analysis, and two were excluded because there was no variation across the selected schools. The median proportions of meals that met the energy recommendations are presented in Table 3. Regarding breakfasts, the presence of a school garden was the only environmental factor significantly associated with energy adequacy. Schools without gardens had significantly (*p* = 0.007) higher median percentages of breakfasts with adequate energy content compared with schools that had gardens (23.7% vs. 16.4%). For lunches, three factors were significantly associated with energy adequacy. Schools in wealthier neighborhoods had a significantly (*p* = 0.024) higher median proportion of lunches with adequate energy content, which decreased in schools in poorer neighborhoods (31.3% vs. 23.8% vs. 21.0%). Schools that provided lunches from an external source had a significantly (*p* = 0.003) higher proportion of meals that met energy requirements than schools that prepared lunches on-site (31.2% vs. 14.5%). Schools that prepared only one meal on-site had no meals within the adequate energy range, and schools that prepared two meals had a significantly (*p* = 0.042) higher proportion of lunches that achieved energy adequacy than those that prepared three meals (31.2% vs. 14.5%). In terms of school snacks, none of the environmental factors examined showed a significant association with energy adequacy at the school level.

## 4. Discussion

The primary aim of this study was to evaluate the nutritional adequacy of school meals in Croatia across 14 public schools in the city of Zagreb using a dataset collected in 2016/2017 and 2017/2018 According to the findings, the majority of school meals did not comply with the energy and macronutrient thresholds derived from the National Guidelines for the Nutrition of Students in Primary Schools [33,34]. When applying a multi-criteria analysis, only three breakfasts, 38 lunches, and one snack met the thresholds for nutritional adequacy. These national guidelines remain regulatory standards, but they need to be updated to reflect current public health issues and advances in nutrition science. Nevertheless, our findings indicate that the primary problem is the effective implementation of these guidelines in schools during the study period. The secondary aim of the study was to examine school environmental factors that may be linked to the energy adequacy of school meals. Because the organization of school food systems has not changed significantly over the past decade, these observations remain relevant today.

The importance of balanced school meals is widely recognized, as they influence children’s growth and shape their eating habits and future food choices [2,3,4,16]. However, the results of this study highlight problems with meal nutritional adequacy in the school food system in Zagreb’s primary schools. More specifically, breakfasts in most schools exceeded the thresholds for energy, protein, and fat, while 80.2% meet the minimum required carbohydrate value. For lunches, the results show the opposite trend: most have lower energy values and rarely reach the minimum threshold for carbohydrates, while protein levels often exceed the threshold. Fat content in lunches varies significantly across schools; in 10 schools it falls below the threshold, whereas in four it exceeds it. The greatest variability was observed in the nutritional adequacy of snacks, where the carbohydrate threshold was met most frequently, followed by energy. In general, regardless of the type of school meal, fat content was among the most restrictive adequacy criteria, followed by energy, as shown in previous studies [11,15,22,23,40]. While breakfast consumption plays an important role in children’s diet quality and their academic performance [41,42,43,44], evidence from international school food systems suggests that systematically monitoring and refining menu planning may, in the long term, enhance the nutritional adequacy of school breakfasts by reducing energy content and limiting foods high in fat, added sugars, and sodium [40,45]. Regarding school lunches, a similar problem with nutritional adequacy has been observed in Slovenia, which has a school food system comparable to ours [22]. Moreover, the same challenge has been reported in other countries, regardless of the country’s income level or serving system [13,15,23]. This nutritional inadequacy of school lunches in the city of Zagreb is even more critical when considered alongside children’s consumption patterns, as they waste more than 20% of the food served at lunch in primary schools [46]. Relatively few countries provide afternoon snacks within school food systems, and these snacks are often low in macronutrients and high in added sugar compared to school lunches. However, when fortified ready-to-eat products are included as part of the snack, they may have higher nutrient density [47]. This suggests that the quality of school snacks depends on the variety and nutrient density of the foods offered. One possible explanation for these observations is that schools in the city of Zagreb did not employ nutritionists to evaluate the nutritional adequacy of prepared menus during the study period. Instead, menu planning was carried out by school staff such as principals, secretaries, kitchen managers, or school chefs. The role of nutritionists and dietitians in the school food system is widely recognized. The Academy of Nutrition and Dietetics, in its statement, supports employing these professionals to plan and oversee school meals [48]. Studies have also shown that schools where nutritionists are involved in menu planning or in developing menu programs serve meals with higher nutritional adequacy [49,50,51]. In the absence of professional nutritional support, meal adequacy depends on the nutrition knowledge, cognitive abilities, and cooking skills of the staff involved in the school food system, which may be limiting factors [25,26,52]. Evaluating school meals solely on energy and macronutrients provides only a partial picture of their quality. A comprehensive assessment should also include additional parameters, such as fiber, sodium, and other micronutrients that are critical during childhood. In addition, assessing meal quality requires considering the distribution and frequency of serving different food groups and the degree of food processing. These meal quality aspects were not directly examined in the present study.

The exploratory bivariate analysis of environmental factors reveals that meal adequacy may be linked to kitchen type, food preparation model, and socioeconomic context of the neighborhood. However, this was observed with energy adequacy in lunches. Schools that received meals from an external source had a higher proportion of meals that met the energy threshold. However, external kitchens typically prepared and delivered only lunch to other schools. Consequently, a school that served as an external kitchen employed more kitchen staff because it prepared a greater number of meals, while schools that prepared all meals independently had, on average, about two to three employees. Because of the statistical methods used, these results are observational and cannot explain how these factors interact to influence the nutritional adequacy of school meals. However, based on on-site observations and dialogs with kitchen staff, it is recommended to distribute kitchen staff’s workload more effectively, taking into account the number of meals prepared and served in each kitchen. Evidence on how kitchen type (central, on-site, or catering) affects meal quality is inconsistent in the existing literature. Most studies report that meal quality is higher in on-site school kitchens or that there is no difference. This suggests that meal quality depends not only on kitchen management and where the food is prepared but also on menu planning [50,51,53]. It is always important to consider the time between meal preparation and serving, especially for meals delivered from an external kitchen, because of nutrient loss [54]. In managing the school food system, financial concerns must be emphasized, which was not the case in this study. Finances may affect kitchen equipment availability, which can, in turn, influence the implementation of national guidelines and the quality of the school food system [25,26,27]. Dialogs with school staff revealed that schools allocate different amounts of money to the school food system (food procurement and kitchen maintenance) and that their kitchens are equipped to varying extents. Most chefs noted that they lacked kitchen aids and equipment that would speed up and facilitate meal preparation (e.g., a blender for preparing homemade cream soups or spreads). One of the research questions was whether the neighborhood poverty status of schools could be associated with meal nutritional adequacy. The results of the present study indicate that schools located in more affluent neighborhoods had a higher median proportion of lunches with adequate energy content. It should be noted that the majority of schools in the city center and some in the suburbs are in more affluent neighborhoods. These results are the opposite of the findings of Ognjenovic et al. (2026), which showed that schools in high-poverty boroughs in London provided more nutritious meals [55]. However, the existing literature also includes studies that found significant differences in the quality of school meals with regard to socioeconomic status [56,57]. Accordingly, the poverty rate may explain some of the differences in the quality of school meals. Participation in various programs that increase the availability of food items such as fruit, vegetables, and dairy products in schools may positively affect the quality of school meals [58,59,60,61]. However, in this study, it was not possible to observe this impact because all schools participated in the School Scheme or Kaufland Fruit and Vegetables programs [36,37]. Finally, school gardens are known to contribute to higher overall diet quality of children and to increased fruit and vegetable intake [62,63]. We observed that schools without gardens provide higher median percentages of breakfasts with adequate energy content. In Croatia, school gardens are used primarily for botanical purposes and nature education rather than as an integrated component of the school food system and nutrition programs. Although the serving times of the meals (breakfast and lunch) did not differ in terms of influencing energy adequacy, it is important to note that, in 50% of the schools included in this research, the meals were served only 2 h apart. Serving two meals within such a short interval can affect children’s satiety and their food consumption, which represents a behavioral risk, especially considering that more than 20% of school lunches are being wasted [46]. These observational findings on environmental factors indicate possible structural reforms. The study shows that on-site kitchens served meals with lower energy adequacy, received less financial support, and had equipment deficits. It is proposed to encourage investment in kitchen infrastructure, improve the distribution of kitchen staff workloads, and enhance their knowledge and cooking skills. Moreover, the study findings highlight the need for menus prepared by qualified professionals and for monitoring guideline implementation in schools. In addition, standardized and frequent audits are needed to detect problems related to guideline implementation in a timely manner. The data for this study were collected within the past decade, but there have been no major changes in the organization of school food systems. The most notable change is that, starting with the 2022/2023 school year, the national government introduced universal school meal funding for children who regularly attend school. This funding usually includes breakfasts. Another change is at the level of the city of Zagreb, where a policy initiative, Green Public Procurement, was launched in 2024/2025 and continues to be implemented in schools. As part of this initiative, schools were offered menus developed by experts, which are being adopted slowly and gradually, depending on each school’s organizational capacity [64,65]. Our findings should be interpreted as a characterization of the school food system during the study period, and policy implications should be considered in light of these recent changes.

One of the major strengths of this work is the inclusion of 14 primary schools from different districts across the city of Zagreb. The analysis of annual menus reduces the impact of seasonal variations and provides insight into regular menu planning. However, certain limitations should be acknowledged. Because the data analysis is limited to the city of Zagreb, generalizing these results to other regions should be done cautiously, although the results offer insights for adjusting school food systems across Croatia. All recipes, including ingredient quantities and preparation methods, were collected from each head chef in each school using the same methodology. However, the nutritional adequacy of meals was assessed only by energy and macronutrient content, without considering other important indicators such as fiber, sodium, and micronutrients. Moreover, the study did not provide an overall measure of meal quality, as it did not examine food group distribution (e.g., fruit and vegetable offerings), the degree of food processing, or meal acceptability. Further research should incorporate these broader indicators when evaluating meal quality. To assess adequacy, we used estimated values based on recommendations for children aged 7–9, who most frequently consume these meals. However, school meals are consumed by children of different ages and sexes, so the assessment does not fully reflect their individual nutritional requirements. Furthermore, this is an analysis of planned and served meals, which does not indicate actual food consumption or energy and nutrient intake by primary school children, especially given the high rate of plate waste in schools [46]. The association between environmental factors and the energy adequacy of meals was assessed using bivariate analyses that did not account for potential confounding factors, so the results should be interpreted with caution and considered exploratory rather than causal. It should also be emphasized that some environmental factors may be interrelated. To address the hierarchical structure of the data, environmental factors were examined at the school level (*N* = 14). Although this aggregate approach is robust and appropriate for school-level analysis, the small sample size limits statistical power. Furthermore, non-parametric analyses may increase the risk of type II errors, potentially obscuring some associations between environmental factors and the energy adequacy of school meals. Nevertheless, the factors were selected based on conditions in the schools and potential problems identified through a review of the existing literature, which still prevail.

## 5. Conclusions

In conclusion, a considerable proportion of school meals in selected primary schools in Zagreb did not meet the recommended energy and macronutrient content during the study period, and the variability between schools highlights an opportunity to better align these meals with existing nutritional guidelines. The observed variability between schools may reflect differences in the implementation of national nutritional guidelines. Among the school-level environmental factors evaluated using exploratory bivariate analyses, kitchen preparation model, number of meals prepared on-site, neighborhood poverty, and presence of a school garden were associated with a higher proportion of school meals that were within the recommended adequate energy range. However, because these environmental factors are interrelated and not adjusted for potential confounders, they should be considered exploratory rather than evidence of a causal effect. These findings indicate the need to improve the nutrient adequacy of school meals, taking into account environmental factors within each school food service system.

## Figures and Tables

**Table 1:** Recommended daily energy and macronutrient intakes and calculated meal-specific thresholds for energy and macronutrients per meal served in primary schools for children aged 7–9 years according to national guidelines.

Variables	Total Daily Intake	Breakfast (15% of Daily Intake)	Lunch (35% of Daily Intake)	Snack (10% of Daily Intake)
Energy (kcal)	1855 (1669–2040)	278 (250–306)	649 (584–714)	186 (167–205)
Protein (g)	58.0 (46.4–69.6)	8.7 (7.0–10.4)	20.3 (16.2–24.4)	5.8 (4.6–7.0)
Fat (g)	67.0 (62.0–72.4)	10.0 (9.3–10.8)	23.5 (21.6–25.3)	6.7 (6.2–7.2)
Carbohydrate (g)	>231.9	>34.8	>81.5	>23.2

**Table 2:** List of selected environmental factors with corresponding variable descriptions.

Factors	Description of the Factor
School location	center, suburb, distant suburb
School neighborhood poverty	poor, average, wealthy
Kitchen type	external, internal
Total number of meal types served	number from 1 to 3
Number of meal types prepared on-site	number from 1 to 3
Time interval between breakfast and lunch ^1^	≤2 h, >2 h
Snack consumption location ^2^	classroom or canteen
Number of kitchen staff	≤2 staff, 3 staff, ≥4 staff
The person who prepares the school menus	nutritionist, other school staff
Participation in the School scheme or Kaufland Fruit and Vegetables ^3^	yes, no
Participation in the Živjeti zdravo program ^4^	yes, no
School garden presence	yes, no

^1^ Analyzed only for breakfast and lunch. ^2^ Analyzed only for snack. ^3^ Programs that provide supplemental procurement of fruit, vegetables, milk, and dairy products. ^4^ An initiative that focused on auditing school menus according to the standards and offering menu changes with accompanying meal recipes.

**Table 3:** Proportion of school meals meeting energy requirements across environmental factors in annual school menus in selected primary schools (*N* = 14) in Zagreb, Croatia.

Environmental Factor	Category	Schools (*n*)	Breakfast Adequate Energy %, Median (IQR)	Lunch Adequate Energy %, Median (IQR)	Snack Adequate Energy %, Median (IQR)
School location	Center	4	15.3 (12.9–18.6)	26.2 (7.2–31.2)	19.7 (12.2–22.4)
	Suburb	6	18.9 (17.9–26.4)	30.6 (11.6–32.4)	20.5 (6.0–27.1)
	Distant suburb	4	22.4 (16.3–24.6)	21.0 (14.5–24.2)	10.2 (8.3–15.5)
	* *p* values		0.265	0.483	0.594
School neighborhood poverty	Poor	5	18.9 (14.3–24.6)	21.0 (4.0–25.4)	10.2 (3.8–23.9)
	Average	3	19.6 (15.9–22.3)	23.8 (12.8–27.8)	18.5 (14.6–19.7)
	Wealthy	4	16.4 (15.2–25.1)	31.3 (29.1–32.7)	21.7 (12.7–26.6)
	* *p* values		*0.971*	*0.024*	*0.509*
Kitchen type	External	6	18.2 (15.3–25.7)	31.2 (27.3–32.2)	21.7 (10.6–27.1)
	Internal	8	19.0 (11.6–23.5)	14.5 (1.3–24.7)	14.4 (5.1–20.7)
	* *p* values		0.662	0.003	0.180
Total number of meal types served	1 meal	1	8.0 (N/A)	0.0 (N/A)	NS
	2 meals	1	19.9 (N/A)	3.9 (N/A)	NS
	3 meals	12	18.6 (15.2–24.4)	25.6 (11.3–31.5)	19.5 (10.1–22.4)
	* *p* values		0.241	0.203	*-*
Number of meal types prepared on-site	1 meal	1	8.0 (N/A)	0.0 (N/A)	NS
	2 meals	7	18.2 (15.3–25.7)	31.2 (27.3–32.2)	21.7 (10.6–27.1)
	3 meals	6	19.0 (11.6–23.5)	14.5 (1.3–24.7)	14.4 (5.1–20.7)
	* *p* values		0.252	0.042	0.150
Time interval between breakfast and lunch ^1^	≤2 h	7	19.3 (16.8–25.7)	29.6 (17.9–32.1)	
	>2 h	7	16.9 (11.6–23.5)	22.4 (6.0–28.6)	
	* *p* values		0.902	0.456	
Snack consumption location ^2^	Canteen	5			18.5 (6.4–20.7)
	Classroom	7			22.9 (10.5–27.1)
	* *p* values				0.202
Number of kitchen staff	≤2 staff	7	18.9 (15.1–26.7)	27.4 (21.0–32.0)	20.7 (10.1–27.0)
	3 staff	5	19.0 (13.7–23.7)	12.8 (0.4–29.8)	14.6 (3.6–20.3)
	≥4 staff	2	12.7 (N/A)	15.3 (N/A)	20.5 (N/A)
	* *p* values		0.334	0.335	0.697
Participation in the Živjeti zdravo program	No	7	16.9 (11.6–27.0)	25.6 (15.8–32.2)	14.4 (5.1–24.0)
	Yes	7	19.3 (16.8–23.1)	26.0 (8.0–30.6)	20.6 (10.6–22.4)
	* *p* values		0.902	0.710	0.589
School garden presence	No	7	23.7 (19.6–26.7)	28.0 (8.0–31.8)	15.7 (9.2–22.5)
	Yes	7	16.4 (11.6–18.5)	23.6 (15.8–30.9)	19.5 (10.2–22.9)
	* *p* values		0.007	0.620	1.000

Data are presented as the median (interquartile range) of the school-level percentages of meals that met the energy recommendation; N/A—the interquartile range was not calculated for subgroups with very small sample sizes (*n* ≤ 2), and these estimates should be considered descriptive and interpreted with caution; NS—meals were not served; “-“—*p* value was not calculated as consumption was identified in only one subgroup; ^1^ Tested only for breakfast and lunch; ^2^ Tested only for snack; * Mann–Whitney U test or Kruskal–Wallis test for comparisons across the groups (*p* < 0.05).

## Data Availability

The data are available upon request from A.I.

## Supplementary Materials

The following supporting information can be downloaded at https://www.mdpi.com/article/10.3390/nu18213709/s1. Table S1: Results of the post hoc analysis of differences in the energy and macronutrient content of breakfasts between 14 primary schools in the city of Zagreb. Table S2: Results of the post hoc analysis of differences in the energy and macronutrient content of lunches between 14 primary schools in the city of Zagreb. Table S3: Results of the post hoc analysis of differences in the energy and macronutrient content of snacks between 14 primary schools in the city of Zagreb.
