# Supplementary material for: Variability in the Nutritional Adequacy of Primary School Meals in Zagreb: The Role of School Environmental Factors
Source: Nutrients. 2026 Nov 9;18(21):3709. doi: 10.3390/nu18213709 (PMC13765277; doi:10.3390/nu18213709)
Supplement: Supplementary file 1 [file nutrients-18-03709-s001.zip › nutrients-4635619-supplementary.pdf]

Supplementary materials

Table S1. Results of the post-hoc analysis of differences in the energy and macronutrient content of breakfasts between 14 primary school in the city of Zagreb

School identity number	p Values*			
School pairs for comparison	Energy	Protein	Fat	Carbohydrate
1 – 2	0.007	0.083	< 0.001	0.661
1 – 3	0.003	0.411	0.302	< 0.001
1 – 4	0.001	0.231	0.001	0.003
1 – 5	0.002	0.016	0.861	< 0.001
1 – 6	0.001	0.009	< 0.001	0.128
1 – 7	< 0.001	< 0.001	0.001	< 0.001
1 – 8	0.232	0.032	0.004	0.059
1 – 9	< 0.001	< 0.001	0.215	< 0.001
1 – 10	0.234	0.029	0.718	0.246
1 – 11	0.023	0.621	< 0.001	< 0.001
1 – 12	< 0.001	0.370	< 0.001	0.283
1 – 13	< 0.001	< 0.001	0.030	< 0.001
1 – 14	< 0.001	0.891	0.564	< 0.001
2 – 3	< 0.001	0.011	< 0.001	< 0.001
2 – 4	0.432	0.595	0.160	0.010
2 – 5	< 0.001	< 0.001	< 0.001	< 0.001
2 – 6	0,525	0.371	0.249	0.277
2 – 7	< 0.001	< 0.001	< 0.001	< 0.001
2 – 8	0.001	< 0.001	< 0.001	0.125
2 – 9	< 0.001	< 0.001	< 0.001	< 0.001
2 – 10	< 0.001	< 0.001	< 0.001	0.110
2 – 11	< 0.001	0.083	0.996	< 0.001
2 – 12	0.565	0.407	0.851	0.523
2 – 13	< 0.001	< 0.001	0.011	< 0.001
2 – 14	< 0.001	0.062	< 0.001	< 0.001
3 – 4	< 0.001	0.043	< 0.001	< 0.001
3 – 5	0.385	0.110	0.392	0.085
3 – 6	< 0.001	0.001	< 0.001	< 0.001
3 – 7	0.031	< 0.001	0.019	0.920
3 – 8	0.233	0.141	0.043	< 0.001
3 – 9	< 0.001	0.001	0.834	< 0.001
3 – 10	0.081	0.170	0.507	0.005
3 – 11	0.508	0.187	< 0.001	< 0.001
3 – 12	< 0.001	0.086	< 0.001	< 0.001
3 – 13	< 0.001	< 0.001	0.001	< 0.001
3 – 14	0.409	0.494	0.650	0.023
4 – 5	< 0.001	< 0.001	0.001	< 0.001
4 – 6	0.881	0.155	0.800	0.142
4 – 7	< 0.001	< 0.001	< 0.001	< 0.001
4 – 8	< 0.001	0.002	< 0.001	0.580

4 – 9	< 0.001	< 0.001	< 0.001	< 0.001
4 – 10	< 0.001	0.001	< 0.001	< 0.001
4 – 11	< 0.001	0.478	0.158	< 0.001
4 – 12	0.836	0.766	0.132	0.056
4 – 13	< 0.001	< 0.001	0.259	< 0.001
4 – 14	< 0.001	0.183	< 0.001	< 0.001
5 – 6	< 0.001	< 0.001	< 0.001	< 0.001
5 – 7	0.052	0.025	0.001	0.067
5 – 8	0.173	0.870	0.006	< 0.001
5 – 9	< 0.001	0.107	0.288	< 0.001
5 – 10	0.051	0.825	0.852	< 0.001
5 – 11	0.384	0.003	< 0.001	0.019
5 – 12	< 0.001	0.001	< 0.001	< 0.001
5 – 13	< 0.001	< 0.001	0.019	< 0.001
5 – 14	0.538	0.023	0.689	0.588
6 – 7	< 0.001	< 0.001	< 0.001	< 0.001
6 – 8	< 0.001	< 0.001	< 0.001	0.519
6 – 9	< 0.001	< 0.001	< 0.001	< 0.001
6 – 10	< 0.001	< 0.001	< 0.001	0.007
6 – 11	< 0.001	0.033	0.247	< 0.001
6 – 12	0.954	0.086	0.182	0.657
6 – 13	< 0.001	< 0.001	0.167	< 0.001
6 – 14	< 0.001	0.006	< 0.001	< 0.001
7 – 8	0.003	0.096	0.909	< 0.001
7 – 9	0.010	0.538	0.033	< 0.001
7 – 10	< 0.001	0.014	0.003	0.006
7 – 11	0.005	< 0.001	< 0.001	< 0.001
7 – 12	< 0.001	< 0.001	< 0.001	< 0.001
7 – 13	< 0.001	< 0.001	< 0.001	< 0.001
7 – 14	0.186	< 0.001	0.005	0.018
8 – 9	< 0.001	0.248	0.064	< 0.001
8 – 10	0.812	0.730	0.010	0.005
8 – 11	0.511	0.011	< 0.001	< 0.001
8 – 12	< 0.001	0.004	< 0.001	0.314
8 – 13	< 0.001	< 0.001	< 0.001	< 0.001
8 – 14	0.062	0.043	0.017	< 0.001
9 – 10	< 0.001	0.067	0.382	< 0.001
9 – 11	< 0.001	< 0.001	< 0.001	0.002
9 – 12	< 0.001	< 0.001	< 0.001	< 0.001
9 – 13	< 0.001	< 0.001	0.001	< 0.001
9 – 14	< 0.001	< 0.001	0.507	< 0.001
10 – 11	0.274	0.007	< 0.001	< 0.001
10 – 12	< 0.001	0.002	< 0.001	0.026
10 – 13	< 0.001	< 0.001	0.012	< 0.001
10 – 14	0.010	0.040	0.831	< 0.001
11 – 12	< 0.001	0.683	0.854	< 0.001
11 – 13	< 0.001	< 0.001	0.011	< 0.001
11 – 14	0.136	0.529	< 0.001	0.073
12 – 13	< 0.001	< 0.001	0.007	< 0.001
12 – 14	< 0.001	0.302	< 0.001	< 0.001
13 – 14	< 0.001	< 0.001	0.001	< 0.001

* p values of Dunn’s post hoc test for pairwise multiple comparisons ($p < 0.05$)

Table S2. Results of the post-hoc analysis of differences in the energy and macronutrient content of lunches between 14 primary school in the city of Zagreb

School identity number	p Values*			
School pairs for comparison	Energy	Protein	Fat	Carbohydrate
1 – 2	0.791	0.789	0.948	0.757
1 – 3	< 0.001	0.022	< 0.001	< 0.001
1 – 4	< 0.001	0.348	0.057	< 0.001
1 – 5	< 0.001	< 0.001	< 0.001	< 0.001
1 – 6	0.031	< 0.001	< 0.001	< 0.001
1 – 7	< 0.001	0.095	< 0.001	0.003
1 – 8	< 0.001	< 0.001	< 0.001	< 0.001
1 – 9	0.837	0.878	0.786	0.958
1 – 10	< 0.001	0.695	0.101	< 0.001
1 – 11	< 0.001	0.122	< 0.001	0.001
1 – 12	< 0.001	0.001	< 0.001	< 0.001
1 – 13	< 0.001	< 0.001	< 0.001	< 0.001
1 – 14	< 0.001	0.336	< 0.001	< 0.001
2 – 3	< 0.001	0.011	< 0.001	< 0.001
2 – 4	< 0.001	0.228	0.049	< 0.001
2 – 5	< 0.001	< 0.001	< 0.001	< 0.001
2 – 6	0.059	< 0.001	< 0.001	< 0.001
2 – 7	< 0.001	0.053	< 0.001	0.001
2 – 8	< 0.001	< 0.001	< 0.001	< 0.001
2 – 9	0.638	0.674	0.712	0.717
2 – 10	< 0.001	0.900	0.008	< 0.001
2 – 11	< 0.001	0.070	< 0.001	< 0.001
2 – 12	< 0.001	< 0.001	< 0.001	< 0.001
2 – 13	< 0.001	0.250	< 0.001	< 0.001
2 – 14	< 0.001	0.219	< 0.001	< 0.001
3 – 4	< 0.001	0.179	< 0.001	0.180
3 – 5	< 0.001	0.002	< 0.001	0.131
3 – 6	< 0.001	< 0.001	< 0.001	< 0.001
3 – 7	< 0.001	0.533	< 0.001	< 0.001
3 – 8	0.013	< 0.001	0.382	0.030
3 – 9	< 0.001	0.033	< 0.001	< 0.001
3 – 10	< 0.001	0.008	< 0.001	< 0.001
3 – 11	< 0.001	0.459	< 0.001	< 0.001
3 – 12	0.155	0.250	0.010	0.060
3 – 13	0.396	< 0.001	< 0.001	0.598
3 – 14	< 0.001	0.187	< 0.001	< 0.001
4 – 5	< 0.001	< 0.001	< 0.001	0.866
4 – 6	< 0.001	< 0.001	< 0.001	< 0.001
4 – 7	< 0.001	0.468	0.004	< 0.001
4 – 8	< 0.001	< 0.001	< 0.001	0.277
4 – 9	< 0.001	0.434	0.108	< 0.001
4 – 10	< 0.001	0.185	0.491	< 0.001
4 – 11	< 0.001	0.545	0.007	< 0.001
4 – 12	0.036	0.013	< 0.001	0.001

4 – 13	< 0.001	< 0.001	< 0.001	0.414
4 – 14	0.019	0.981	< 0.001	< 0.001
5 – 6	< 0.001	< 0.001	< 0.001	< 0.001
5 – 7	< 0.001	< 0.001	< 0.001	< 0.001
5 – 8	0.278	0.013	< 0.001	0.342
5 – 9	< 0.001	< 0.001	< 0.001	< 0.001
5 – 10	< 0.001	< 0.001	< 0.001	< 0.001
5 – 11	< 0.001	< 0.001	< 0.001	< 0.001
5 – 12	< 0.001	0.045	< 0.001	0.001
5 – 13	< 0.001	0.118	< 0.001	0.324
5 – 14	< 0.001	< 0.001	< 0.001	< 0.001
6 – 7	< 0.001	< 0.001	< 0.001	0.431
6 – 8	< 0.001	< 0.001	< 0.001	< 0.001
6 – 9	0.019	< 0.001	< 0.001	< 0.001
6 – 10	< 0.001	< 0.001	< 0.001	0.316
6 – 11	< 0.001	< 0.001	< 0.001	0.790
6 – 12	< 0.001	< 0.001	< 0.001	< 0.001
6 – 13	< 0.001	< 0.001	< 0.001	< 0.001
6 – 14	< 0.001	< 0.001	< 0.001	0.002
7 – 8	< 0.001	< 0.001	< 0.001	< 0.001
7 – 9	< 0.001	0.131	< 0.001	0.004
7 – 10	0.745	0.040	0.030	0.073
7 – 11	0.901	0.906	0.855	0.602
7 – 12	< 0.001	0.076	< 0.001	< 0.001
7 – 13	< 0.001	< 0.001	0.012	< 0.001
7 – 14	< 0.001	0.483	0.481	< 0.001
8 – 9	< 0.001	< 0.001	< 0.001	< 0.001
8 – 10	< 0.001	< 0.001	< 0.001	< 0.001
8 – 11	< 0.001	< 0.001	< 0.001	< 0.001
8 – 12	< 0.001	< 0.001	0.003	< 0.001
8 – 13	< 0.001	0.224	< 0.001	0.080
8 – 14	< 0.001	< 0.001	< 0.001	< 0.001
9 – 10	< 0.001	0.586	0.022	< 0.001
9 – 11	< 0.001	0.165	< 0.001	0.001
9 – 12	< 0.001	0.001	< 0.001	< 0.001
9 – 13	< 0.001	< 0.001	< 0.001	< 0.001
9 – 14	< 0.001	0.420	< 0.001	< 0.001
10 – 11	0.655	0.054	0.047	0.203
10 – 12	< 0.001	< 0.001	< 0.001	< 0.001
10 – 13	< 0.001	< 0.001	< 0.001	< 0.001
10 – 14	< 0.001	0.178	0.004	0.033
11 – 12	< 0.001	0.059	< 0.001	< 0.001
11 – 13	< 0.001	< 0.001	0.007	< 0.001
11 – 14	0.015	0.561	0.376	0.001
12 – 13	0.562	< 0.001	0.267	0.016
12 – 14	< 0.001	0.014	0.004	< 0.001
13 – 14	< 0.001	< 0.001	0.074	< 0.001

* p values of Dunn’s post hoc test for pairwise multiple comparisons ($p < 0.05$)

Table S3. Results of the post-hoc analysis of differences in the energy and macronutrient content of snacks between 14 primary school in the city of Zagreb

School identity number	p Values*			
School pairs for comparison	Energy	Protein	Fat	Carbohydrate
1 – 2	< 0.001	0.652	< 0.001	0.021
1 – 4	< 0.001	< 0.001	< 0.001	< 0.001
1 – 5	< 0.001	0.007	< 0.001	< 0.001
1 – 6	< 0.001	< 0.001	< 0.001	< 0.001
1 – 7	< 0.001	0.086	< 0.001	0.007
1 – 9	< 0.001	0.166	< 0.001	0.008
1 – 10	< 0.001	< 0.001	< 0.001	< 0.001
1 – 11	< 0.001	< 0.001	< 0.001	< 0.001
1 – 12	< 0.001	0.065	< 0.001	< 0.001
1 – 13	< 0.001	0.146	< 0.001	0.002
1 – 14	0.001	0.769	< 0.001	0.244
2 – 4	< 0.001	< 0.001	< 0.001	0.001
2 – 5	0.022	0.002	0.010	0.043
2 – 6	< 0.001	< 0.001	0.005	< 0.001
2 – 7	0.194	0.030	0.149	0.682
2 – 9	0.891	0.066	0.274	0.717
2 – 10	< 0.001	< 0.001	< 0.001	< 0.001
2 – 11	< 0.001	< 0.001	0.846	< 0.001
2 – 12	< 0.001	0.022	0.085	< 0.001
2 – 13	0.495	0.056	0.301	0.458
2 – 14	0.797	0.457	0.762	0.257
4 – 5	0.082	0.017	0.310	0.241
4 – 6	0.185	0.128	0.461	0.002
4 – 7	0.006	0.001	0.033	0.005
4 – 9	< 0.001	< 0.001	< 0.001	0.005
4 – 10	0.534	0.144	0.928	0.375
4 – 11	< 0.001	< 0.001	0.001	< 0.001
4 – 12	0.975	0.001	0.065	0.237
4 – 13	0.001	< 0.001	< 0.001	0.014
4 – 14	< 0.001	< 0.001	0.001	< 0.001
5 – 6	0.002	0.393	0.785	< 0.001
5 – 7	0.322	0.333	0.264	0.106
5 – 9	0.032	0.195	< 0.001	0.097
5 – 10	0.019	0.356	0.271	0.040
5 – 11	< 0.001	< 0.001	0.018	< 0.001
5 – 12	0.077	0.404	0.405	0.019
5 – 13	0.108	0.217	< 0.001	0.198
5 – 14	0.011	0.017	0.024	0.002
6 – 7	< 0.001	0.069	0.167	< 0.001
6 – 9	< 0.001	0.032	< 0.001	< 0.001
6 – 10	0.483	0.948	0.410	0.028
6 – 11	< 0.001	< 0.001	0.008	< 0.001
6 – 12	0.197	0.093	0.271	0.056
6 – 13	< 0.001	0.037	< 0.001	< 0.001

6 – 14	< 0.001	0.001	0.012	< 0.001
7 – 9	0.246	0.741	0.011	0.741
7 – 10	0.001	0.059	0.027	< 0.001
7 – 11	< 0.001	< 0.001	0.210	< 0.001
7 – 12	0.006	0.897	0.779	< 0.001
7 – 13	0.537	0.790	0.013	0.741
7 – 14	0.121	0.156	0.255	0.123
9 – 10	< 0.001	0.027	< 0.001	< 0.001
9 – 11	< 0.001	< 0.001	0.197	< 0.001
9 – 12	< 0.001	0.646	0.005	< 0.001
9 – 13	0.585	0.948	0.950	0.706
9 – 14	0.694	0.276	0.164	0.136
10 – 11	< 0.001	< 0.001	0.001	< 0.001
10 – 12	0.556	0.080	0.054	0.769
10 – 13	< 0.001	0.031	< 0.001	0.001
10 – 14	< 0.001	0.001	0.001	< 0.001
11 – 12	< 0.001	< 0.001	0.126	< 0.001
11 – 13	< 0.001	< 0.001	0.218	< 0.001
11 – 14	< 0.001	< 0.001	0.913	< 0.001
12 – 13	0.001	0.693	0.006	< 0.001
12 – 14	< 0.001	0.122	0.157	< 0.001
13 – 14	0.348	0.247	0.182	0.061

* p values of Dunn's post hoc test for pairwise multiple comparisons ($p < 0.05$)
